# Optimizing use of theranostic nanoparticles as a life-saving strategy for treating COVID-19 patients

**DOI:** 10.7150/thno.46691

**Published:** 2020-05-01

**Authors:** Rasha Itani, Mansour Tobaiqy, Achraf Al Faraj

**Affiliations:** 1 Department of Radiologic Sciences, Faculty of Health Sciences, American University of Science and Technology (AUST), Beirut, Lebanon; 2 Department of Pharmacology, College of Medicine, University of Jeddah, Jeddah, Saudi Arabia

**Keywords:** COVID-19, drug delivery systems, nanomedicine, theranostic nanoparticles

## Abstract

On the 30^th^ of January 2020, the World Health Organization fired up the sirens against a fast spreading infectious disease caused by a newly discovered Severe Acute Respiratory Syndrome Coronavirus 2 (SARS-CoV-2) and gave this disease the name COVID-19. While there is currently no specific treatment for COVID-19, several off label drugs approved for other indications are being investigated in clinical trials across the globe.

In the last decade, theranostic nanoparticles were reported as promising tool for efficiently and selectively deliver therapeutic moieties (i.e. drugs, vaccines, siRNA, peptide) to target sites of infection. In addition, they allow monitoring infectious sides and treatment responses using noninvasive imaging modalities. While intranasal delivery was proposed as the preferred administration route for therapeutic agents against viral pulmonary diseases, NP-based delivery systems offer numerous benefits to overcome challenges associated with mucosal administration, and ensure that these agents achieve a concentration that is many times higher than expected in the targeted sites of infection while limiting side effects on normal cells.

In this article, we have shed light on the promising role of nanoparticles as effective carriers for therapeutics or immune modulators to help in fighting against COVID-19.

## Introduction

**Figure 1:**
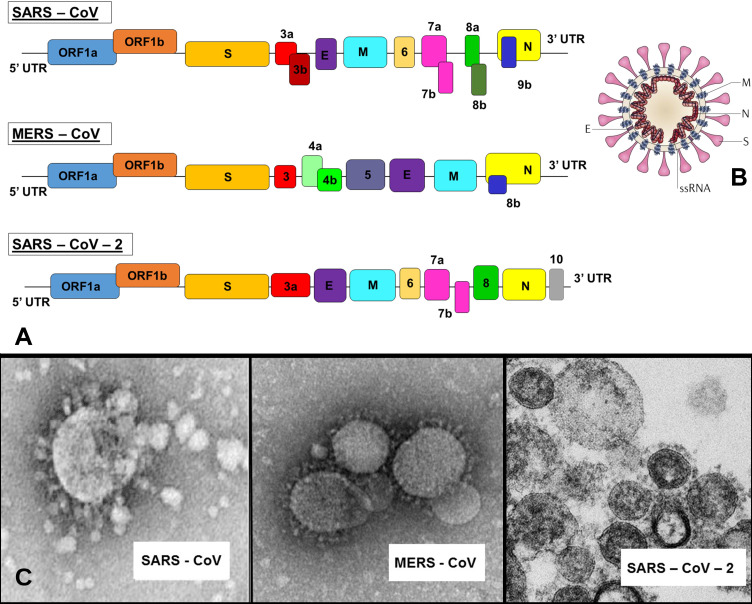
Single-stranded RNA (ssRNA) genome of 27-34 kb for SARS, MERS and novel SARS-2 coronaviruses (A) with a schematic of the coronavirus's structure: enveloped and spherical particle of around 125 nm in diameter (B) along with respective Transmission Electron Microscopy (TEM) images (C). Adapted from 7 and Centers for Disease Control and Prevention (CDC) database.

Coronaviruses are a large group of viruses that belong to the Coronaviridae family. They were named for the crown-like spikes on their surface, and were reported to cause diseases in humans and few animal species with wide spectrum of severity. To date, four main sub-groupings of coronaviruses have been identified named alpha, beta, gamma, and delta 1. Considered one of the largest among known RNA viruses, the genome size of coronaviruses, which are enveloped with a positive-sense single-stranded RNA genome and a nucleocapsid of helical symmetry, ranges from approximately 27 to 34 kilobases with a diameter of around 125 nm 2. The first known severe illness in humans caused by a coronavirus emerged in 2003 in China and resulted in the Severe Acute Respiratory Syndrome (SARS) epidemic 3. A second outbreak of severe infection occurred in 2012 in Saudi Arabia and led to the Middle East Respiratory Syndrome (MERS) 4. A novel strain of coronavirus causing severe illness was recently reported in December 2019 in Wuhan, China and was subsequently named SARS-CoV-2 **(Figure 1)**. On 30 January 2020, the World Health Organization (WHO) Emergency Committee announced a global health emergency based on significant number of confirmed cases and on 11 February 2020 gave the disease an official name: COVID-19 (which stands for COrona Virus Infectious Disease - 2019). The virus has raised world concern because of its high transmission rate as well as high mobility and mortality 5. The number of confirmed cases is increasing daily and can be tracked in almost real time on the website of the WHO website 6.

Person-to-person transmission of SARS-CoV-2 is expected to occur mainly via respiratory droplets produced during coughing, sneezing and talking, and largely resembles the spread of influenza 8. However, transmission can occur when an infected person, with or without symptoms, are in close contact with a healthy one, or when one touches an infected surface and then touches his or her eyes, nose, or mouth 9. The period between infection and symptom onset might range from 2 to 14 days 10. It is mainly believed that SARS-CoV-2 droplets do not typically travel for more than 2 meters and do not remain in the air. However, Van Doremalen N. et al. reported, under experimental conditions, that SARS-CoV-2 aerosols can remain viable for up to 3 hours in aerosols, 4 hours on copper, 24 hours on cardboard and 2 to 3 days on plastic and stainless steel 11.

Studies have shown that patients with underlying diseases such as hypertension, lung disease, and cardiovascular disease may have higher mortality risk than other patients 12, 13. Another study suggested that other risk factors related to developing Acute Respiratory Distress Syndrome (ARDS) and progressing to death include: age, neutrophilia, organ failure, and coagulation dysfunction 14. However, these studies are still limited due to the lack of sufficient information about this novel COVID-19 disease and the limited number of patients included in these studies.

This article will first review the proposed conventional treatments that are currently under extensive research and clinical trials. Then, the advantages of theranostic nanoparticles with a special focus on the optimal formulations for intranasal administration of the various therapeutic agents will be discussed. Finally, a special focus will be devoted to the development of nanoparticles-based treatment modalities that are projected to considerably improve COVID-19 therapy.

## Proposed Conventional Treatments

Currently, there are no validated vaccines or specific antiviral treatments for COVID-19. Most treatments currently used including cardiovascular/ hemodynamic or respiratory are supportive, that is, they support patients suffering from the virus. Unfortunately, these treatments are given to relieve complications and side effects, but do not efficiently kill the virus. Therefore, extensive research and clinical trials evaluating potential therapy are still imminently necessary.

To develop a successful treatment for COVID-19, one must understand well the mechanism of action of the virus. Resembling SARS and MERS coronaviruses, this novel SARS-CoV-2 uses a “Lock and Key” mechanism in which the angiotensin converting enzyme II (ACE2) acts as a “key” to enter specialized cells holding its “lock” 13. These target sites can be found in the lungs, heart, arteries, kidneys, and intestines cells. Once inside, the virus will use the host cell's organelles to replicate and infect other cells. Based on that, a treatment that prevents the virus's entry into the cell may be of benefit.

What is common about all target cells for SARS-CoV-2 is the AP2-associated protein kinase 1 (AAK1), a key regulator of endocytosis. Richardson P. et al. proposed, using a machine learning software BenevolentAI, that drugs associated with AAK1 may suppress viral access into the target cells 15. However, high doses of these inhibitors such as oncology drugs (Sunitinib and Erlotinib) will be required but could unfortunately lead to serious side effects 16. Furthermore, the simulations also showed that not all AAK1 inhibitors could cause severe side effects. For example, Baricitinib, a janus kinase (JAK) inhibitor, can bind to another endocytosis regulator, cyclin G-associated kinase, and inhibit AAK1, thus preventing the virus entry into the cell. In addition to being used in cases of rheumatoid arthritis, it can be investigated as a potential treatment to fight against COVID-19 17.

Other potential inhibitors to fight COVID-19 include the complementary use of human immunodeficiency virus (HIV) protease inhibitors such as Lopinavir and Ritonavirin that have been shown to suppress the 3-chymotrypsin-like protease of SARS and MERS 18. Several phase III and phase IV clinical trials have been initiated to assess the efficacy of these antiviral drugs. For instance, phase IV open, prospective/retrospective, randomized controlled cohort study was designed to assess the efficacy of Lopinavir/Ritonavirin antiviral drugs in the treatment of COVID-19 viral pneumonia (NCT04255017). A clinical study is projected to evaluate the safety and effectiveness of Baricitinib vs. Lopinavir/ritonavir (along with 2 other medications) in hospitalized persons with moderate to severe COVID-19 disease (NCT04321993).

Other suggested alternative treatments under extensive investigation include virally targeted agents, mainly Remdesivir, a nucleoside analogue that targets the RNA-dependent polymerase and suppresses viral RNA synthesis in a broad spectrum of RNA viruses, including human coronaviruses. Remdesivir is an approved HIV reverse transcriptase inhibitor that has shown broad-spectrum activities against RNA coronaviruses in cell cultures and animal models 19, 20. Holshue M. et al. reported the successful recovery of a SARS-CoV-2 infected patient receiving intravenous administration of Remdesivir with no adverse events 21. To further assess its safety and efficacy, several phase 3 clinical trials were initiated in patients with COVID-19 (NCT04292899, NCT04292730, and NCT04252664).

A very promising treatment option that started to be applied in several countries involved the use of Chloroquine or Hydroxy-Chloroquine, antiviral and wide spread drugs previously used against malaria and autoimmune diseases 22. A protocol comprising the use of Hydroxy-Chloroquine reinforced by azithromycin revealed encouraging outcomes for efficient treatment of COVID-19. However, potential effectiveness was mainly observed in the early impairment of contagiousness, and treatment should be administered under close monitoring of physicians due to concerns about the risk of arrhythmic death. A Chloroquine-based drug was reported to inhibit the fusion of SARS coronavirus with the cells by acidifying the lysosomes and thus inhibiting catherpsins that require a low pH for optimal cleavage of SARS-CoV-2 spike protein 23. It is assumed that Chloroquine can either alter the molecular crosstalk of SARS-CoV-2 with its target cells through suppression of kinases (i.e. MAPK), or interfere with proteolytic processing of the M protein and affect virion assembly and budding. Furthermore, Chloroquine can indirectly act by reducing the production of pro-inflammatory cytokines and/or by activating anti-SARS-CoV-2 CD8+ T-cells 24. Cortegiani A. et al. have recently reviewed the efficacy and safety of Chloroquine for the treatment of COVID-19 25.

However, despite these proposed treatment options currently under extensive research and clinical trials, the death toll of patients infected with this novel coronavirus is still increasing. Therefore, efforts should in parallel focus on alternative approaches in order to achieve an effective treatment while minimizing side effects. Although mucosal vaccination, mostly intranasal, is the preferred route of vaccination against infectious diseases, treatment modalities currently under investigation use the systemic route. This is mainly because intranasal treatment needs to overcome many hurdles, such as low intrinsic permeability for some drugs, limited volume of administration, rapid mucociliary clearance, and enzymatic degradation, before they reach the targeting site due to the unique characteristics of the mucosal environment. Therefore, the delivery of most proposed therapies to specific sites of the body by means of nanoparticles (NP) ensures that these agents achieve a concentration several times higher than those obtained via conventional methods in the targeted sites of infection while limiting any side effects on normal cells.

## Intranasal Delivery of Theranostic Nanoparticles

In the last decade, theranostic nanoparticles have emerged as a new field of medicine combining specific targeted therapy based on diagnostic tools for the next generation treatment of several diseases. The low toxicity, added to the size, charge, and chemical modification capabilities of these nanoparticles, allow them to overcome the multiple barriers that impede their way following various administration routes. More specifically, extensive efforts have focused on the development of a NP-based intranasal delivery system as an effective and safe tool to deliver several therapeutic moieties (i.e. vaccine, drugs, siRNA, peptide, antibodies, etc.) 26. Importantly, NP delivery systems offer numerous benefits for mucosal administration and include i) protecting the therapeutic moieties against enzyme degradation; ii) extending their residence and release time; iii) ensuring their co-delivery with adjuvants; iv) increasing the concentration of conjugated materials in target cells; v) offering receptor-ligand mediated targeting delivery; and vi) potentiating the immune system at the same time 27.

Mucosal treatment is highly desired for infectious diseases since most pathogens initiate their infections at the human mucosal surface. Intranasal delivery allows for noninvasive, practical, simple, and inexpensive administration of therapeutic agents. The large surface area and rich capillary plexuses also allow for their quick absorption 28. These administration routes have already been assessed for vaccination against respiratory viruses such as influenza and coronaviruses 29.

While the nanoparticles' pharmacokinetic properties are mainly governed by their characteristics, the shape, size and surface charge of the designed nanocarriers are considered as crucial factors that should be taken into consideration when optimized for intranasal delivery and thus play a crucial role in the success of the treatment. Several studies were performed to identify the optimal characteristics of the theranostic nanoparticles for pulmonary intranasal administration and were recently reviewed 30. It was concluded that an optimal lung delivery system is expected to have a size smaller than 100-200 nm for enhanced immune responses, display a slight positive charge to improve cell-association, be synthesized with a mixture of NP-loaded and surface-conjugated therapeutic moieties, while displaying sufficient hydrophobicity.

It is worth noting that majority of studies performed to assess the use of nanoparticles as delivery system following intranasal pulmonary administration are mostly based on preclinical data performed on small animal and cannot be readily generalized to humans. However, the investigations provide some promising forecasts for prospective clinical applications with theranostic nanoparticles.

**Figure 2:**
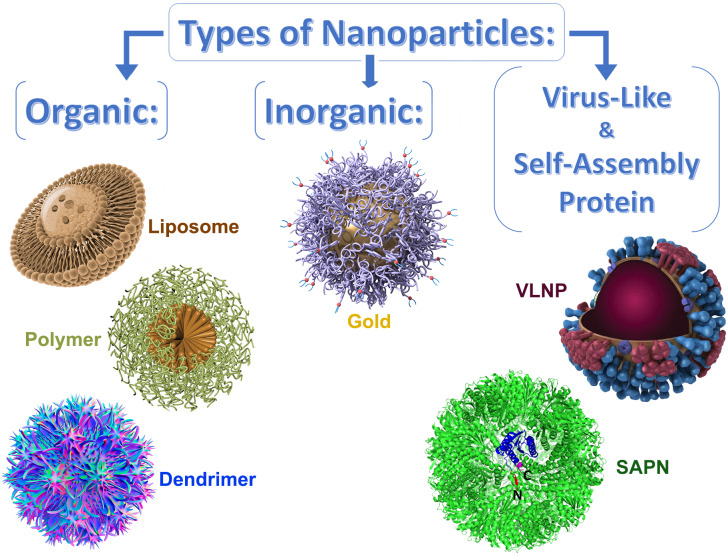
Schematic design of the various types of nanoparticles-based delivery systems that can be optimized for intranasal pulmonary administration of therapeutic agents.

Several types of theranostic nanoparticles were proposed as promising for intranasal administration. They can be divided into 3 broad categories: organic, inorganic, and virus-like or self-assembling protein nanoparticles **(Figure 2)**.

### Organic Nanoparticles

#### Lipid Nanoparticles

Nanoparticles made from lipids are particularly attractive for biomedical applications owing to their enhanced biocompatibility imparted by the lipid material. Among the various lipid-based formulations adapted for intranasal delivery are liposomes, which are spherical capsules having an outer phospholipid bilayer and an inner hydrophilic core designed to hold aqueous therapeutic agents 31. Liposomes offer numerous advantages including efficient encapsulation of the conjugated agents and simple modification to further enhance their mucosal and cellular uptake and improve their biocompatibility 32. Like any other type of nanoparticles, surface charge plays an important role in affecting the pharmacokinetic properties of liposomes. In fact, studies carried on cationic liposomes following intranasal administration showed higher absorption and enhanced bioavailability compared to their negatively charged counterparts. This is due to the negative charge of the mucosal membranes leading to electrostatic attraction of these positively charged nanoparticles as well as reducing their clearance by the mucosal cilia 33. Furthermore, liposomes were reported to have great potential for mucosal vaccinations as their retention in the nasal cavity induces a high immune activity leading to the production of higher levels of immunoglobulins 34.

#### Polymer Nanoparticles

Polymer-based nanoparticles were reported as an attractive delivery system mainly due to the possibility of tailoring their properties and functions to a specific application. They can be synthesized by the addition of several monomers into various configurations including linear, branched, and 3D networks (i.e. highly branched) 35, and thus their size, shape, and surface charge can be easily optimized to allow a controlled release of their cargo under external conditions 36. Out of the many formulations of polymer nanoparticles, those made of Chitosan attracted particular interest for intranasal administration due to their nontoxic nature, biocompatibility, biodegradability into non-toxic products *in vivo*, capability to open up tight junctions between epithelial cells 37, and ability to be easily modified into desired shapes and sizes 38. Upon conjugation with therapeutic compounds, Chitosan can enhance persistence of polymeric NP in mucosal environment and penetration to mucosal tissue.

#### Dendrimer Nanoparticles

Dendrimer are radially symmetric molecules with well defined, homogeneous, and monodisperse structure. Similar to Polymer, Dendrimer NP can be synthetized in highly branched 3D networks with greater ability of attaching many functional groups on their surface, and encapsulating non-water soluble, hydrophobic therapeutic agents in their core 39. This allows the potential use of these nanoparticles in various therapies against tumors, bacterial and viral infections 40.

With the strong interactions that they make with viruses, dendrimers showed enhanced antiviral activities, preventing the infection of the host. Consequently, they became an important tool in the treatment of viral infections such as HIV and influenza virus infections 41.

Nandy B. et al. reported the development of Poly-L-lysine (PLL)-based dendrimeric nanoparticles with anionic naphthalene disulphonate surface that can block the entry of HIV viruses by binding to the viral envelope protein gp120 and preventing the formation of the CD4-gp120 complex 42. Chahal J. et al. developed dendrimer NP encapsulating an antigen-expressing replicon mRNA. This nanoformulation offered vital CD8+ T-cell and antibody responses that can efficiently protect against lethal exposures to several deadly pathogens, including Ebola, H1N1 influenza, and Toxoplasma gondii pathogens 43.

### Inorganic Nanoparticles

Engineered inorganic nanoparticles are attracting special interests due to their ability to not only act as conventional delivery systems to efficiently deliver loaded cargo to target sites, but also to allow stimuli-responsive characteristics and intrinsic capability of some types (i.e. Magnetic or Gold Nanoparticles) to be monitored following *in vivo* administration to human body using noninvasive medical imaging 44. While inorganic NP are extensively investigated in preclinical and clinical studies for the detection, diagnosis and treatment of many diseases, some concerns are still arising about their safe clinical applications 45. To overcome this, researchers are working on functionalizing inorganic NP with various types of biocompatible materials thus offering the potential benefits of both organic and inorganic nanoparticles.

Gold nanoparticles have shown special interest in vaccine development as they can easily trigger the immune system via internalization by antigen presenting cells. The synthesis methodologies, significant progress, and future prospects of the use of Gold NP for new mucosal vaccines were reviewed 46. Gold nanoparticles can be easily adapted and customized for intranasal delivery and can have the advantage of being readily diffused into lymph nodes thus activating CD8+ (T-killer) cell-mediated immune response 47. Furthermore, Gold nanoparticles, owing to their high atomic number, can also function as excellent highly stable and biocompatible contrast agent for X-ray based medical imaging, especially in Computed Tomography (CT) 48.

### Virus-like and Self-assembling Protein Nanoparticles

Virus-like NP (VLNP) are sphere-shaped nanoparticles composed of several molecules with sizes ranging between 20 and 200 nm. These nanoparticles result from the self-assembly of proteins derived from viral capsids. They were introduced as attractive nanomaterials as they do not contain genetic material but have the ability for accurately mimicking the real virus or antigen in terms of structure and antigenic determinant(s). This makes these nanomaterials highly attractive to antigen presenting cells that can be readily identified and consequently can trigger an immune response 49. Studies performed following intranasal delivery of VLNP derived from the influenza virus lead to enhancing the immunity against this virus by triggering significant types of immune responses (cellular and humoral). Hence, they act as a vaccine that can prevent further infections (i.e. influenza virus) by producing a significantly high amount of antibodies and T-cells 50. Another advantage of VLNP is their high potential to be used as a vector in gene therapy, where they can be used as a smart system to accurately deliver a transgene to the site of the mutation or repair genes in aim of changing gene expression or encoding a protein 51. Moreover, VLNP's promising treatment rely on the fact that they can easily prevent enzymatic degradation compared to the naked administration of viroids (viral DNA segments) and they have extremely small size allowing their penetration into the cellular nucleus 52. Interestingly, these innovative VLNP can be also adapted to be detected using various noninvasive medical imaging modalities (i.e. MRI and PET) and thus offering a theranostic platform for next-generation diagnosis and treatment of viral infections 53.

Self-assembling protein nanoparticles (SAPN) are novel type of NP obtained from the oligomerization of monomeric proteins with a dimeter ranging from 20 to 100 nm. Self-assembly is defined as the autonomous organization of molecules into a more stable structure by using non-covalent bonding mechanisms to achieve equilibrium 54. It has paved the way for developing robust and functional NP for various applications. These nanoparticles can be engineered using many biomaterials with peptides being the most favorable due to the fact that these protein-based NP can be easily developed and modified for numerous applications 55. They were assessed in drug delivery given their distinguishing ability to cross the cellular membrane and specifically and safely deliver drugs, genes and nucleic acids directly to the cell's nucleus 56.

Kanekiyo M. et al. reported the synthesis of SAPN that elicit broader and more effective immunity (i.e. tenfold higher haemagglutination inhibition antibody titres) than traditional influenza vaccines following intranasal inoculation, and thus provide a promising platform for developing broader vaccine protection against emerging viruses and other pathogens 57.

## Nanoparticles-based Treatment Modalities

The use of NP in the medical field holds great promise in developing novel theranostic and diagnostic solutions for treating COVID-19. It is out of the scope of this review to discuss the innovative solutions that NP can offer in fighting against coronavirus such as the development of disinfecting spray that have the ability to kill the virus on the surface, or detection tools (i.e. rapid screening test).

**Figure 3:**
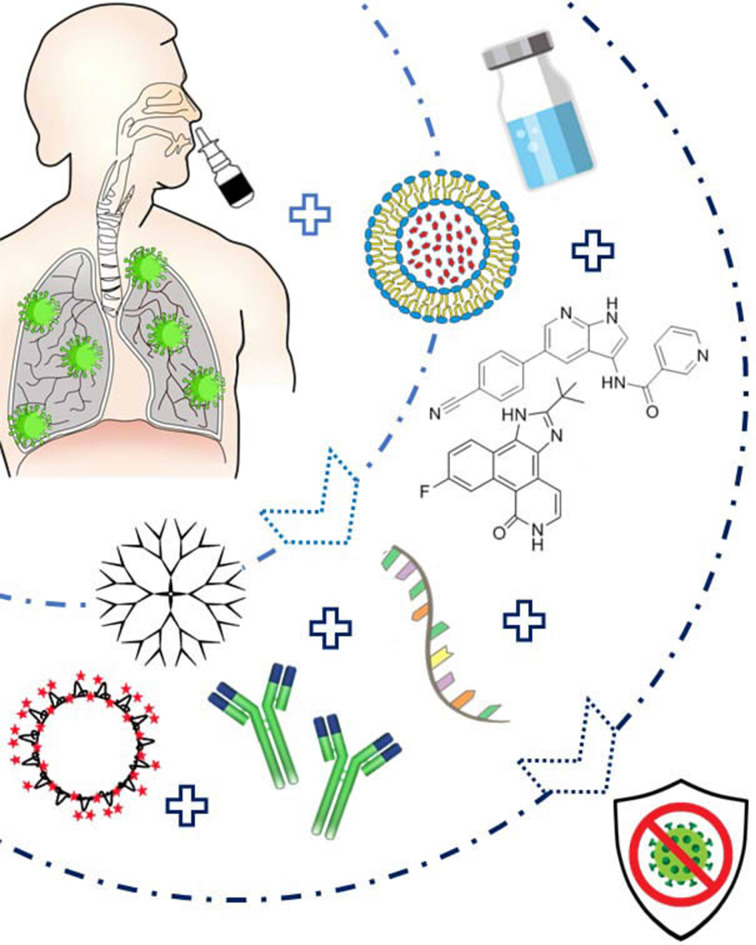
Diagram presenting the suggested Nanoparticles-based treatment approach that can be delivered via intranasal route. The nanoparticles can be either conjugated to therapeutic agents such as specific siRNA, peptide inhibitors (AAKI and JAK), or antibodies, or can administered as virus-like NP. They are prepared into an emulsion or solution, and easily administered to the patients via a nasal spray thus leading to an efficient therapy against SARS-CoV-2.

As SARS-CoV-2 has a diameter of around 125 nm fitting in the nanoscale size range, biocompatible theranostic NP can thus be very promising to simultaneously detect and neutralize this novel coronavirus by several approaches as previously investigated against various viral infections including SARS or MERS coronaviruses **(Figure 3).**

### Enhanced Drug Delivery

As previously mentioned, drugs associated with AAK1 may suppress virus access into the cells. However, high doses of these suppressors such as oncology drugs (Sunitinib and Erlotinib) will be required but could unfortunately lead to serious side effects. Therefore, the use of NP as smart nanocarriers that can selectively enhance the delivery of such drugs to target cells will certainly reduce the required doses and arising side effects while providing a more efficient treatment.

Drug Delivery System (DDS) conjugated with either Sunitinib 58, 59 or Erlotinib 60 were extensively assessed for treatment of cancerous diseases. As an example, Xu H. et al. investigated a DDS-based on Erlotinib-conjugated liposomes for treatment of lung cancer. Results showed that this nanoformulation can considerably enhance the drug targeting, improve the drug biodistribution following *in vivo* administration in the body (i.e. 3 months stability), and significantly increase the relative bioavailability of the drug (i.e. fast and sustained release within the first 4 hours) 61. Once such oncology drugs that are associated with AAK1 are validated to effectively suppress viral access into the target cells in COVID-19, NP formulations conjugated with these drugs, and optimized for either intranasal or intravenous delivery, are expected to provide positive outcomes for COVID-19 treatment.

### Specific siRNA Delivery

Since coronaviruses are positive ssRNA viruses that use ORF1a and ORF1b replicases, RNA interference (RNAi) could be an efficient approach to control the virus by silencing the viral mRNA at particular stages in human cells 62. RNAi technology inhibits the expression of the homologous sequence in a target RNA by incorporating small non-coding RNAs into cells to a multi-protein RNA-induced silencing complex (RISC). After identifying the sequence to be inhibited, small interfering RNAs (siRNA) will be separated from RISC and link to that specific section 63. Complementary strands of appropriate mRNA will then get degraded using special enzymes. Therefore, siRNAs can play a crucial role in regulating virus infections and replication 64.

siRNA were reported as a very successful candidate to fight against viruses as they can be identified, designed, and synthesized to attack every possible viral mutation 65. Several studies reported the efficacy of siRNA in inhibiting viral replications such as in Hepatitis C virus (HCV) 63. However, one of the essential factors that lead to the success of this potential treatment is their specific and selective delivery to target sites. The administration of naked or unprotected siRNA will be potentially exposed to degradation by enzymes, and lead to several side effects including toxicity, instability, and filtration by kidneys and reticuloendothelial system (RES) 66. Therefore, optimizing specific nanocarriers to deliver this fragile molecule to its target is essential.

Sohrab S. et al. recently reviewed the design and delivery of therapeutic siRNAs for treating MERS-Coronavirus 65. The delivery of siRNAs was enhanced by using novel nanocarriers such as lipid, inorganic, or polymeric nanoparticles.

The effective delivery of siRNA via lipid nanoparticles revealed promising results against several viral diseases 67. Various formulations of lipid nanoparticles (LNP) such as liposomes, solid, semisolid, or liquid state, or nano-emulsions were evaluated. LNP protect siRNA from nucleases, improve their biodistribution, ensure selective delivery to the desired sites, and enhance the bioavailability of therapeutic compounds with low solubility 68.

Inorganic nanoparticles such as gold, magnetic iron oxide, and silica nanoparticles, in addition to quantum dots (QDs) and carbon nanotubes (CNTs) were evaluated as potential carriers for siRNA 69. Gold NP emerged as attractive nanocarriers and were investigated as siRNA carrier and target-specific gene silencing against viral and cancerous diseases owing to their unmatched biocompatibility, flexible configurations and surface modifications, and effective delivery mechanism 70.

Polymeric nanoparticles, either natural (i.e. chitosan) or synthetic (i.e. polyethyleneimine (PEI)), have been also assessed as drug or gene carriers and can be potentially used to specifically deliver siRNA in COVID-19 cases. Chitosan-based NP have proven efficient in inducing protective immunity against various infectious diseases 37 and have been investigated in the formulation of various vaccines including HBV vaccines 71, Newcastle disease vaccines 72, and DNA vaccines 73.

### Peptide Inhibitors

As previously discussed, knowing SARS-CoV-2 infection mechanism will help developing a successful treatment. Luckily, MERS-CoV and SARS-CoV-2 share the same membrane protein (the “key”) which is the Spike Protein (S protein). Therefore, both viruses infect host cells using the S protein mediated fusion between their membranes 74. The S protein, which is the base for MERS infections, contains 2 subunits S1 and S2 that control the host cell's binding to the virus through dipeptidyl peptidase 4 (DPP4) receptor 75. Therefore, inhibiting this fusion will offer a promising approach to similarly treat SARS-CoV-2.

Huang X. et al. proposed the use of Pregnancy Induced Hypertension (PIH) as powerful heptad repeat 1 (HR1) peptide inhibitor that suppresses HR1/HR2-mediated membrane fusion between MERS coronavirus and host cells as the key pathway of MERS-induced host infections. They showed that this peptide inhibitor when delivered via gold nanorods revealed 10 times enhanced inhibitory activity when compared to free PIH 76. Moreover, this nanocomplex showed high stability and biocompatibility with promising prospective application for MERS treatment and similar coronaviruses.

### Prevention of Coronaviruses Entry into Cells

With the continuous evolution of new and mutated strands of viruses, the development of effective and safe antiviral treatments to that specific strain becomes more challenging due to the variation in their genetic compositions and the need for specific antiviral agents. Theranostic nanoparticles can also play a major role in, not only killing the virus inside the body, but also preventing the virus entry into the cells. Owing to their high specific surface area and the ability to adhere to multiple antigens and/or compounds on their surface, nanomaterials such as gold NP and Carbon Quantum Dots (CQDs) were reported as promising tools for interacting with viruses and preventing their entry into cells 77.

A study carried on Boronic acid conjugated Carbon dots NP demonstrated efficiency and success in inhibiting HIV entry by suppressing syncytium formation 78. In another interesting study, Łoczechin A. et al. showed that Boronic acid ligands conjugated with CQDs interfered with the function of coronavirus S protein, and considerably stopped its entry into the host cells 79. It was reported that the addition of these NP to the cell culture medium, before and during infection with coronavirus, considerably reduced the infection rate of the cells. Remarkably, after one viral life cycle (i.e. 5.5 hours for coronavirus), a great inhibition activity was also detected at the viral replication step. These nanomaterials with an average diameter of 10 nm and excellent water solubility showed as promising candidates for winning the battle against coronavirus, because they easily enter the cell through endocytosis and interact with the virus's protein, thereby preventing viral genome replication.

### Stimulation of Cells' Immune System using Virus-like Nanoparticles

With the significant advances in vaccine development, great interest and huge efforts have focused on developing vaccines that mimic the virus using virus-like nanoparticles (VLNP). NP were reported to enhance transport in the lymphatic system compared to smaller subunit antigens 80. Moreover, NP have the ability to display several antigens on their surface facilitating the stimulation of the immune system compared to antigen presenting cells (APCs) that can only present one type of antigen on their surface 81. This has shed the light on the importance of NP not only as carriers of therapeutic material, but also as efficient stimulants of the body's immune system, thus having a double, synergetic function.

NP are known for their high surface energy that leads to strong adhesion of biomolecules 82. This ability can be exploited in order to imitate viral features and characteristics efficiently and thus stimulate the immune system to produce antibodies and immune cells to fight viral infections 83.

In a study combining 100 nm gold NP with the S protein of Infectious Bronchitis Virus (IBV), Chen H. et al. reported increased stability when using the developed VLNP, as well as significant retention of these S proteins (about 900 S proteins per particle) compared to viral antigens 84. Moreover, this study emphasized the delivery enhancing abilities of gold NP compared to free circulating compounds, especially in the lymphatic system where the strong adhesion between NP and the S protein increased the delivery 6 times. Most importantly, it was concluded that these VLNP resulted in the synthesis of higher IgG levels due to the enhanced delivery by the gold NP that lead to increased uptake by cells and intensified complement activation 84.

In another study, Coleman C. et al. reported protection induced by vaccination with a recombinant MERS-CoV S NP vaccine and Matrix-M1 adjuvant combination that were able to efficiently and completely block MERS-CoV replication in the lungs of mice 85. The MERS-CoV S NP vaccine produced high titer anti-S neutralizing antibody and protected against MERS-CoV infection *in vivo* in mice. These studies proved the promising advantage of NP conjugated with S protein as a potential and successful vaccine, to not only stimulate the immune system, but also to protect humans from MERS-CoV, and thus can be applied to SARS-CoV-2 as both coronaviruses have the same key (i.e. Spike protein).

## Conclusions and Perspectives

The highly contagious novel coronavirus SARS-CoV-2 that has infected so far more than 2 million people in 210 countries triggered an unprecedented economic crisis as a consequence of forced lockdown to limit the transmission, and put the life of many infected people at high risk around the world. COVID-19 patients are mainly managed with supportive care that includes cardiovascular/ hemodynamic or respiratory procedures. While there is currently no specific treatment for COVID-19, several drugs approved for other indications are being investigated in clinical trials. These treatments are based on the administration of agents that either block the virus entry inside the host cells stopping virus replication and infection of other cells, or potentially inhibit protease activity (i.e. lopinavir/ ritonavirin antiviral drugs). Other potential treatments under clinical investigations use either nucleoside analogues that target the RNA-dependent polymerase suppressing viral RNA synthesis (i.e. Remdesivir), or directly act on the virion assembly and budding and alter the molecular crosstalk of SARS-CoV-2 while indirectly reducing the production of pro-inflammatory cytokines and/or activating anti-SARS-CoV-2 CD8+ T-cells (i.e. (Hydroxy)-Chloroquine). However, despite some promising results, the death toll of infected patients is still increasing.

Although the administration of proposed conventional treatment is mainly performed via intravenous route, as intranasal administration faces several challenges associated with mucosal environment, the use of Nanoparticle-based delivery system ensures effective treatment while minimizing side effects of therapeutic agents. The theranostic nanoparticles ensure that therapeutic moieties such as drugs, vaccines, siRNA and peptide achieve a concentration that is many times higher than expected in the targeted sites while protecting the therapeutic agents from enzyme degradation. Several types of theranostic nanoparticles that can be divided in 3 broad categories organic (i.e. lipid, polymer, dendrimer), inorganic (i.e. gold), and virus-like or self-assembling protein nanoparticles, were investigated for intranasal administration. The shape, size and surface charge of the designed nanocarriers are considered as crucial factors that should be taken into consideration when optimized for intranasal delivery and thus play a crucial role in the success of the treatment.

Delivered via intranasal route, biocompatible theranostic nanoparticles can thus be a very promising approach to fight against this novel SARS- CoV-2 as previously investigated against various viral infections including SARS or MERS coronaviruses using several approaches. Theranostic nanoparticles can enhance the delivery of therapeutic drugs, ensure selective and specific delivery of siRNA, efficiently delivery peptide inhibitors, prevent coronavirus entry into cells, and stimulate cells' immune system.

Investigating the various promising therapeutic agents currently under development against SARS-CoV-2, delivered via biocompatible theranostic nanoparticles via intranasal route is expected to be far more efficient than any other treatment for COVID-19 treatment.
